# Loss of SNAP29 Impairs Endocytic Recycling and Cell Motility

**DOI:** 10.1371/journal.pone.0009759

**Published:** 2010-03-18

**Authors:** Debora Rapaport, Yevgenia Lugassy, Eli Sprecher, Mia Horowitz

**Affiliations:** 1 Department of Cell Research and Immunology, Tel Aviv University, Ramat Aviv, Israel; 2 Tel Aviv University, Ramat Aviv, Israel; 3 Department of Dermatology, Tel Aviv Sourasky Medical Center, Tel Aviv, Israel; 4 Center for Translational Genetics, Rappaport Institute and Technion – Israel Institute of Technology, Haifa, Israel; University of Birmingham, United Kingdom

## Abstract

Intracellular membrane trafficking depends on the ordered formation and consumption of transport intermediates and requires that membranes fuse with each other in a tightly regulated and highly specific manner. Membrane anchored SNAREs assemble into SNARE complexes that bring membranes together to promote fusion. SNAP29 is a ubiquitous synaptosomal-associated SNARE protein. It interacts with several syntaxins and with the EH domain containing protein EHD1. Loss of functional SNAP29 results in CEDNIK syndrome (Cerebral Dysgenesis, Neuropathy, Ichthyosis and Keratoderma). Using fibroblast cell lines derived from CEDNIK patients, we show that SNAP29 mediates endocytic recycling of transferrin and β1-integrin. Impaired β1-integrin recycling affected cell motility, as reflected by changes in cell spreading and wound healing. No major changes were detected in exocytosis of VSVG protein from the Golgi apparatus, although the Golgi system acquired a dispersed morphology in SNAP29 deficient cells. Our results emphasize the importance of SNAP29 mediated membrane fusion in endocytic recycling and consequently, in cell motility.

## Introduction

In eukaryotic cells, intracellular protein trafficking is based on vesicular transport in which cargo molecules are transferred from “donor” compartments to targeted specific “acceptor” compartments. This complex transport requires vesicle budding and fusion [1]. The fusion process involves SNAREs (Soluble NSF Attachment Protein Receptors or “SNAP receptors”), which comprise two main families of conserved membrane-associated proteins: the v-SNAREs (vesicular) VAMP/synaptobrevins and the t-SNAREs (target) syntaxins and SNAPs [2]. Transport vesicles carry a specific v-SNARE that binds to cognate t-SNAREs to form a trans-SNARE complex (SNAREpin), which becomes a cis-SNARE complex in the fused membrane [3]. The stable cis-SNARE core complex is subsequently dissociated by the action of α-SNAP and the ATPase N-ethylmaleimide-sensitive factor (NSF) [4]. SNAREs perform two major functions: they promote vesicle fusion and ensure the specificity of the process.

The SNAP family of t-SNAREs contains four members: SNAP23, SNAP25, SNAP29 and SNAP47. SNAP25 participates in the synaptic SNARE complex, mediating synaptic vesicle fusion and exocytosis [5]. SNAP23, the non-neuronal homolog of SNAP25, is enriched in platelets and is required for exocytosis [6]. SNAP47 is also a neuronal SNAP showing a widespread distribution on intracellular membranes of neurons and it is enriched in synaptic vesicle fractions. *In vitro*, SNAP47 substituted for SNAP25 in SNARE complex formation with the neuronal SNARE syntaxin 1A and synaptobrevin 2 [7]. SNAP29 is localized predominantly to intracellular membrane structures, which partially overlap with endosomal, lysosomal and Golgi markers [8]. The human SNAP29 is 83% identical to its rat ortholog, GS32 [9]. It is expressed in non-neuronal cells and interacts with most members of the syntaxin family. SNAP29 has been proposed to be a ubiquitous SNARE protein, involved in general membrane trafficking [2]. SNAP29 is also present at synapses, interacts with syntaxin 1A, competes with α-SNAP for binding to the SNARE complex and consequently modulates synaptic transmission, by inhibiting disassembly of the SNARE complex [10]. Overexpression of SNAP29 in presynaptic neurons inhibited synaptic transmission, causing a defect in synaptic vesicle turnover by inhibiting disassembly of the SNARE complex. Accordingly, knockdown of SNAP29 expression in neurons by RNAi increased the efficiency of synaptic transmission, suggesting that SNAP29 acts as a negative modulator for neurotransmitter release, probably by slowing recycling of the SNARE based fusion machinery and synaptic vesicle turnover [11].

SNAP29 forms complexes with clathrin, α-adaptin of adaptor protein 2 (AP2) and EHD1, a member of the EH (Esp15 homology) domain containing protein family, indicating its involvement in the endocytic machinery [12] and has recently been shown to interact with the GTPase Rab3A in myelinating glia [13].

We have recently characterized a novel neurocutaneous syndrome that we termed CEDNIK syndrome for CErebral Dysgenesis, Neuropathy, Ichthyosis and Keratoderma. CEDNIK syndrome was found to be caused by a 1-bp deletion in the SNAP29 gene, resulting in the absence of the protein. All CEDNIK patients examined so far exhibit severe psychomotor retardation as well as generalized ichthyosis (scaling). Additional signs include microcephaly and facial dysmorphism, hypoplastic optic disk, sensorineural deafness and severe cachexia. Of interest, brain MRI showed various degrees of corpus callosum dysgenesis as well as cortical dysplasia, with pachygyria and polimicrogyria indicative of defective neuronal migration. Markedly abnormal epidermal cell differentiation was found to underlie the skin phenotype in CEDNIK syndrome. SNAP29 deficiency was found to prevent both the maturation and the secretion of lamellar granules, which are Golgi-derived vesicular structures responsible for the transport and transfer of lipids and proteases to the upper layers of the epidermis [14].

In order to gain insight into the biological role of SNAP29, we explored its involvement in endocytic and exocytic processes. While exocytosis of VSVG protein was not affected, endocytic recycling of transferrin and β1-integrin was impaired in CEDNIK cells, affecting cell motility and migration.

## Results

### Structure of SNAP29

**Figure 1:**
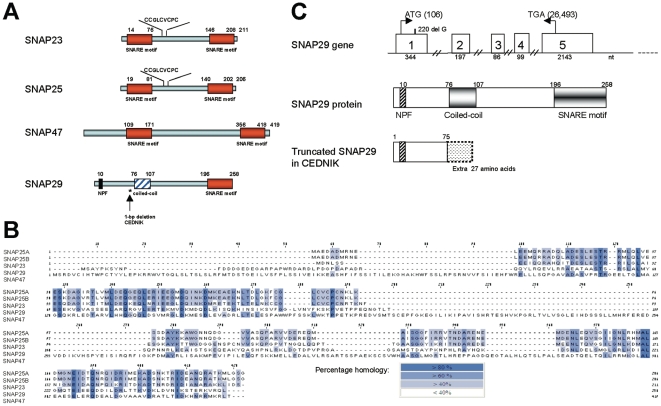
The SNAP family. (A) Domain organization of the SNAP family members: SNAP23, SNAP25, SNAP47 and SNAP29 (modified from: [7]). The arrow and the asterisk indicate a 1-bp deletion at nucleotide 222 of the cDNA, starting from the ATG (c.222delG amino acid 75) in CEDNIK patients. Shown in SNAP23 and SNAP25 is the cysteine (C) rich motif at the central part of the protein, which serves as a palmitoylation signal. Red box represents the SNARE motif, dashed blue box represents a coiled-coil domain and black box represents the NPF motif. Upper numbers in SNAP29 protein represent amino acids. Truncated SNAP29 protein is represented by premature termination 27 amino acids downstream the mutation. (B) Multiple alignment of human SNAP family proteins (SNAP25A and B isoforms, SNAP23, SNAP29 and SNAP47). Accession numbers are as follows: hSNAP25A (P60880); hSNAP25B (Q53EM2) [54]; hSNAP23 (O00161); hSNAP29 (O95721) and hSNAP47 (Q5SQN1). Sequences were aligned using ClustalW (http://www.ebi.ac.uk/clustalw) and BoxShade softwares (http://www.ch.embnet.org/software/BOX_form.html). Blue boxes indicate the percentage homology. (C) The SNAP29 gene, the normal and the truncated (CEDNIK) proteins. In the gene: white boxes represent the exons (1–5) with their respective nucleotide size under the boxes. ATG and TGA are defined by their genomic nucleotide numbers (in parenthesis). The site of the G deletion in CEDNIK is indicated. In the protein: motifs are defined by their amino acid numbers, a.a.- amino acids; NPF- aspargine-proline-phenylalanine; nt- nucleotides.

SNAP family contains four known members in mammals: SNAP23, SNAP25, SNAP29 and SNAP47 (Figure 1A and B). They mediate membrane fusion during intracellular trafficking by forming a four helices bundle, in which one helix is contributed by the v-SNARE (VAMP/synaptobrevin) and three by the t-SNARES (SNAP and syntaxin). Of the three t-SNARE helices, one originates from syntaxin and two originate from the SNAP member [1]. Indeed, SNAP23, SNAP25 and SNAP47 contain two SNARE motifs, which assemble into coiled-coil structures, while SNAP29 has one SNARE motif and a coiled-coil domain, which most probably comprises the second helix (Figure 1A) [15]. In order to induce membrane fusion, SNAP proteins must interact or embed in the membrane, despite the absence of a transmembrane domain. SNAP23 and SNAP25 anchor to the target membrane through post-translational palmitoylation in one or more cysteins found in the central part of these proteins (Figure 1A). SNAP29, as well as its closest homolog SNAP47 [7], lacks a conserved stretch of cysteine residues and any membrane anchor motif (Figure 1A and B). In contrast to other members of the family, SNAP29 has an amino acid stretch with a coiled-coil structure and a N-terminal asparigine-proline-phenylalanine (NPF) protein binding motif (Figure 1A and C), shown to interact with the EH domain of EHD1 [9]. The SNAP29 gene comprises 5 exons. In CEDNIK patients, a one bp deletion in exon 1 (G at cDNA position 222, starting from the ATG) [14] leads to a complete absence of the protein due to premature termination, 27 amino acids downstream the mutation (Figure 1C).

### Normal expression and localization of EHD proteins in the absence of SNAP29

**Figure 2:**
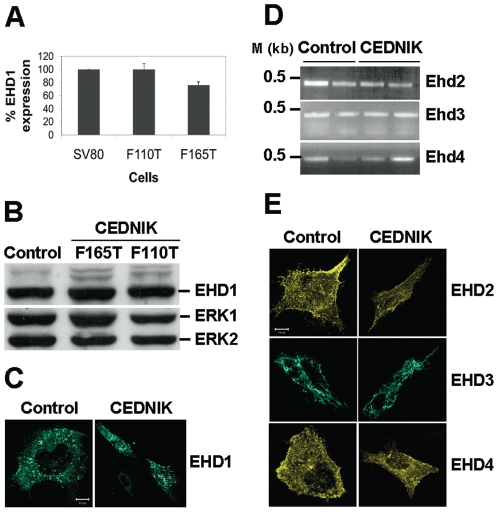
Expression of EHDs in CEDNIK fibroblasts. (A) RNA was isolated from control (SV80) and CEDNIK (F110T and F165T) fibroblasts and was subjected to Ehd1 specific quantitative real-time PCR (qRT-PCR). Expression level of Ehd1 in SV80 was defined as 100%. (B) Protein lysates prepared from CEDNIK (F165T, F110T) and control (SV80) fibroblasts were subjected to Western blot analysis and interacted with anti-EHD1 and anti-ERK antibodies, as a loading control. (C) Fibroblasts (SV80 and F110T) were transfected with GFP-EHD1, fixed and visualized by confocal microscopy. (D) RNA was isolated from SV80 and CEDNIK fibroblasts and subjected to Ehd2, Ehd3 or Ehd4 specific RT-PCR as detailed under Materials and Methods. (E) Control (SV80) and CEDNIK (F110T) fibroblasts were transfected with YFP-EHD2, GFP-EHD3 and YFP-EHD4 expressing plasmids for 18 h, after which they were fixed and visualized by confocal microscopy. No change in EHD1 RNA level, protein level or localization was observed in CEDNIK cells, compared to control cells. Also, no change in the intracellular localization or RNA levels of other EHDs was observed in CEDNIK cells. M- 1 kb marker. Bar,10 µm.

SNAP29 interacts with EHD1 [12], a membrane associated protein, which mediates endocytic recycling [16], [17], [18]. To determine whether absence of SNAP29 affects EHD1, we tested its expression level and intracellular localization in cells lacking SNAP29. The results (Figure 2) showed that neither the RNA level of EHD1, tested by quantitative real-time PCR (Figure 2A), nor its protein level (Figure 2B), were changed in CEDNIK cells. Its punctate distribution in endosomal vesicles in CEDNIK fibroblasts resembled that in control cells (Figure 2C) [19], [20]. Since previous yeast two hybrid results indicated possible interaction of SNAP29 with other members of the EHD family [12], we also tested the expression level and intracellular localization of other EHDs in the absence of SNAP29. The results indicated that neither RNA levels (Figure 2D), tested by RT-PCR, nor the intracellular localization of any transfected EHD (Figure 2E) were altered in CEDNIK cells in comparison to control cells. EHD2 presented a typical membranal staining [21], [22], [23], EHD3 appeared in tubular-vesicular structures [24], [25] and EHD4 showed membranal and endocytic staining (Figure 2E) [26].

### VSVG secretory pathway is normal in SNAP29 deficient cells despite altered Golgi morphology

**Figure 3:**
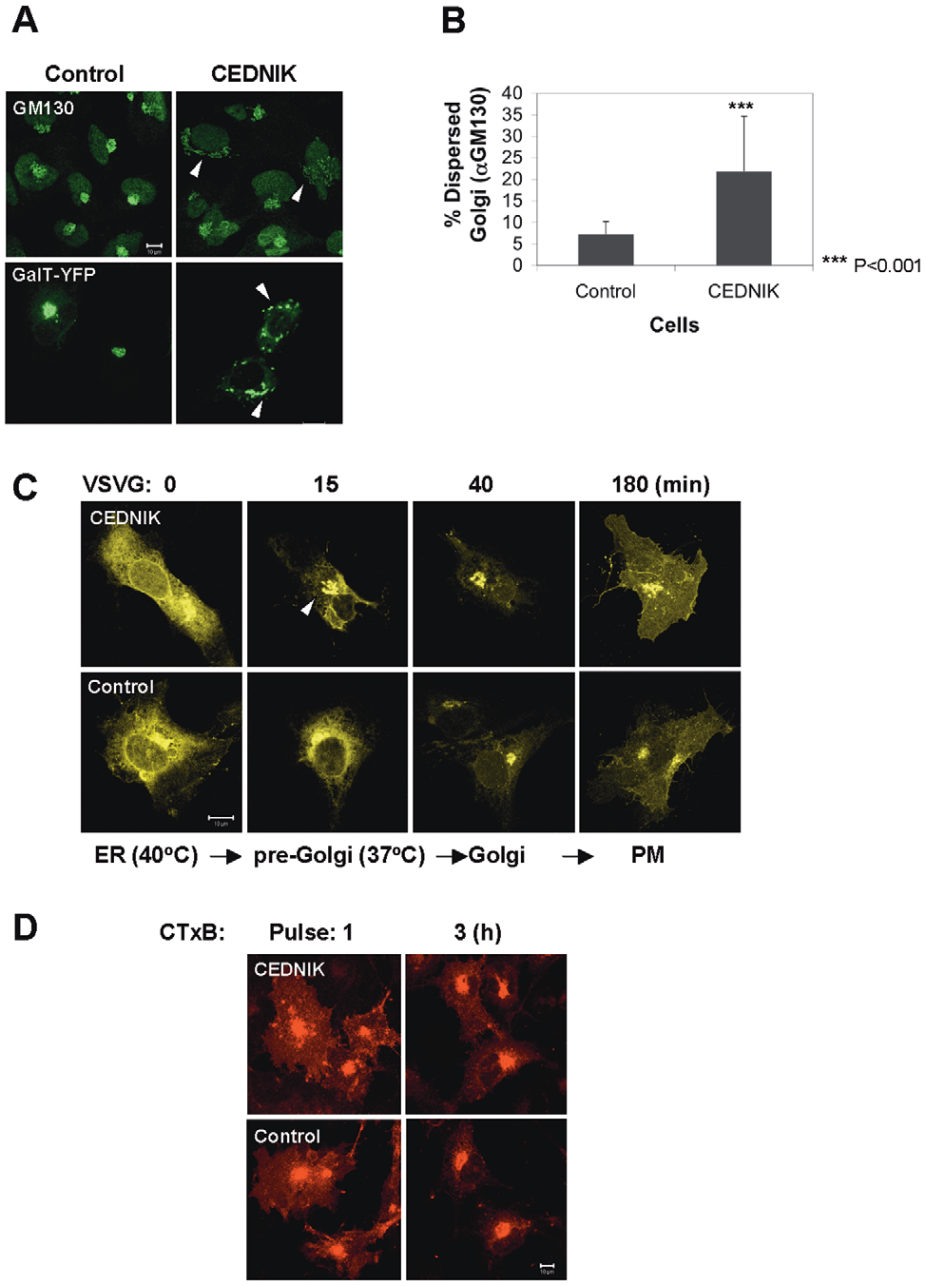
Altered Golgi morphology, intact exocytosis and trafficking to the Golgi in CEDNIK fibroblasts. (A) CEDNIK (F110T) and control (SV80 or F572T) fibroblasts were either transfected with a GalT-YFP expressing plasmid or stained with anti-GM130 antibodies. Arrows point to fragmented and dispersed Golgi. (B) Dispersed versus perinuclear Golgi pattern was counted in 500–750 randomly chosen cells, stained with anti-GM130 antibodies. Asterisks indicate statistical significance of ***p<0.001 as analyzed by the Student's *t*-test. (C) CEDNIK (F110T) and control (SV80) cells were transfected with a VSVG-YFP expressing plasmid and grown at 40°C for 18 h. Cells were shifted to 37°C at the indicated times, fixed and visualized by confocal microscopy. The arrow indicates the atypical Golgi in CEDNIK cells. (D) Cells, labeled with AlexaFluor 555-conjugated CTxB, for the indicated times, were fixed and visualized by confocal microscopy. Golgi complex appeared dispersed and fragmented in CEDNIK cells. VSVG trafficking from the Golgi complex to the plasma membrane (PM) and CTxB trafficking from the PM to the Golgi appeared intact in CEDNIK cells. Bar, 10 µm.

The most striking ultrastructural abnormality in the epidermis of CEDNIK patients was the presence of empty lamellar granules in the spinous and granular epidermal layers, which were not found in control epidermis. Lamellar granules of various sizes and contents were also abnormally present in the lower layers of the significantly thickened stratum corneum in patients' skin but not in normal skin [14]. Since lamellar granules derive from the Golgi apparatus, this morphological abnormality prompted us to test whether the morphology and function of the Golgi apparatus are affected by the loss of SNAP29. A significant fraction of CEDNIK fibroblasts, stained with antibodies against the cis-Golgi protein GM130, presented a dispersed and fragmented cisternae (Figure 3A). The phenomenon was similar in CEDNIK cells transfected with β-(1,3) galactosyltransferase (GalT, a Golgi enzyme marker) expressing plasmid (Figure 3A). Quantitation indicated that while 22% of CEDNIK cells had a fragmented/dispersed Golgi, only 7% of control fibroblasts showed altered morphology (Figure 3B).

We next examined the functional efficacy of the secretory pathway in CEDNIK cells. For this purpose, we followed the transport of a Vesicular Stomatitis Virus (VSV) protein from the Golgi complex to the plasma membrane. The tested protein was a temperature sensitive variant of the VSVG protein (VSVG-ts045). This mutant protein misfolds and is retained in the endoplasmic reticulum (ER) at 40°C, but upon temperature shift to a lower temperature it moves to the pre-Golgi/Golgi complex before being transported to the plasma membrane. Albeit the dispersed morphology of the Golgi apparatus in CEDNIK fibroblasts, the delivery of VSVG from the ER to the plasma membrane was similar to that in control cells (Figure 3C).

Since delivery from the Golgi to the plasma membrane was intact, we analyzed the trafficking from the plasma membrane to the Golgi, using the B subunit of cholera toxin (CTxB). Cholera toxin, which binds GM1 on the plasma membrane, internalizes via clathrin-dependent and -independent mechanisms [27]. It does not recycle, but reaches the Golgi apparatus, from where it traffics to the cytoplasm through the ER. The results showed that trafficking of the B subunit of cholera toxin from the plasma membrane to the Golgi apparatus was intact in CEDNIK cells (Figure 3D).

### Intracellular localization of endocytic markers is altered in SNAP29 depleted cells

**Figure 4:**
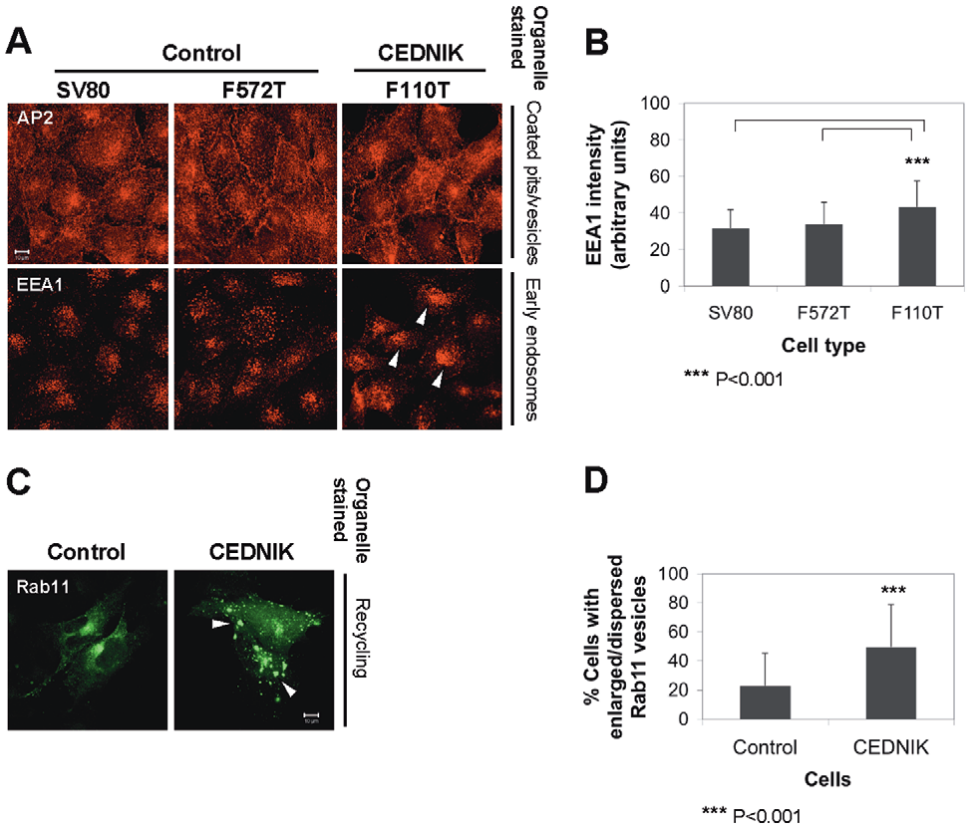
Changes in morphology of endocytic vesicles in SNAP29 deficient cells. Control (SV80 and F572T) and CEDNIK (F110T) fibroblasts were fixed and stained with (A) anti-AP2 and anti-EEA1 antibodies. Arrows indicate the intense accumulation of EEA1 positive endosomes in CEDNIK fibroblasts. (B) Bar graph shows quantitation of the intensity of EEA1 staining (presented in arbitrary units), measured in 90–100 cells, using the ImageJ software. (C) Control (SV80) and CEDNIK (F110T) fibroblasts were transfected with Rab11-YFP expressing plasmid, fixed and visualized by confocal microscopy. Arrows indicate Rab11 positive enlarged vesicles. (D) Bar graph shows quantitation of the enlarged and dispersed Rab11 positive vesicles in CEDNIK (F110T) in comparison to control (SV80) cells. Bar,10 µm. Asterisks indicate statistical significance of ***p<0.001 as analyzed by the Student's *t*-test. EEA1 intensity is significantly stronger in CEDNIK compared to control cells. Some Rab11 domains are dispersed.

Aiming at understanding the cellular abnormalities in CEDNIK cells, we followed the morphology of organelles associated with intracellular trafficking and endocytic processes that require SNARE-mediated membrane fusion (Figure 4). In CEDNIK cells, AP2, a marker of coated pits and coated vesicles, showed a typical membrane and punctate cytosolic distribution, respectively (Figure 4A). Early endosomes, stained with anti-EEA1 (early endosome antigen 1 protein) antibody, showed a punctate dispersed vesicle staining in control cells, while in CEDNIK cells, staining was of a higher intensity (Figure 4 A and B), reflecting accumulation of early endosomes due to their impaired ability to fuse. Rab11, a marker of the endocytic recycling endosomes, was found in a well defined perinuclear compartment in control cells, while an increased number of enlarged dispersed vesicles was observed in CEDNIK cells, with a less defined perinuclear localization (Figure 4C). This aberrant pattern was found in 50% of CEDNIK fibroblasts and in only 23% of control fibroblasts (Figure 4D). These results confirmed the impaired ability of vesicles to fuse properly in CEDNIK cells and strongly indicated that SNAP29 mediated fusion regulates several steps of endocytosis.

### Transferrin recycling is attenuated in CEDNIK cells

**Figure 5:**
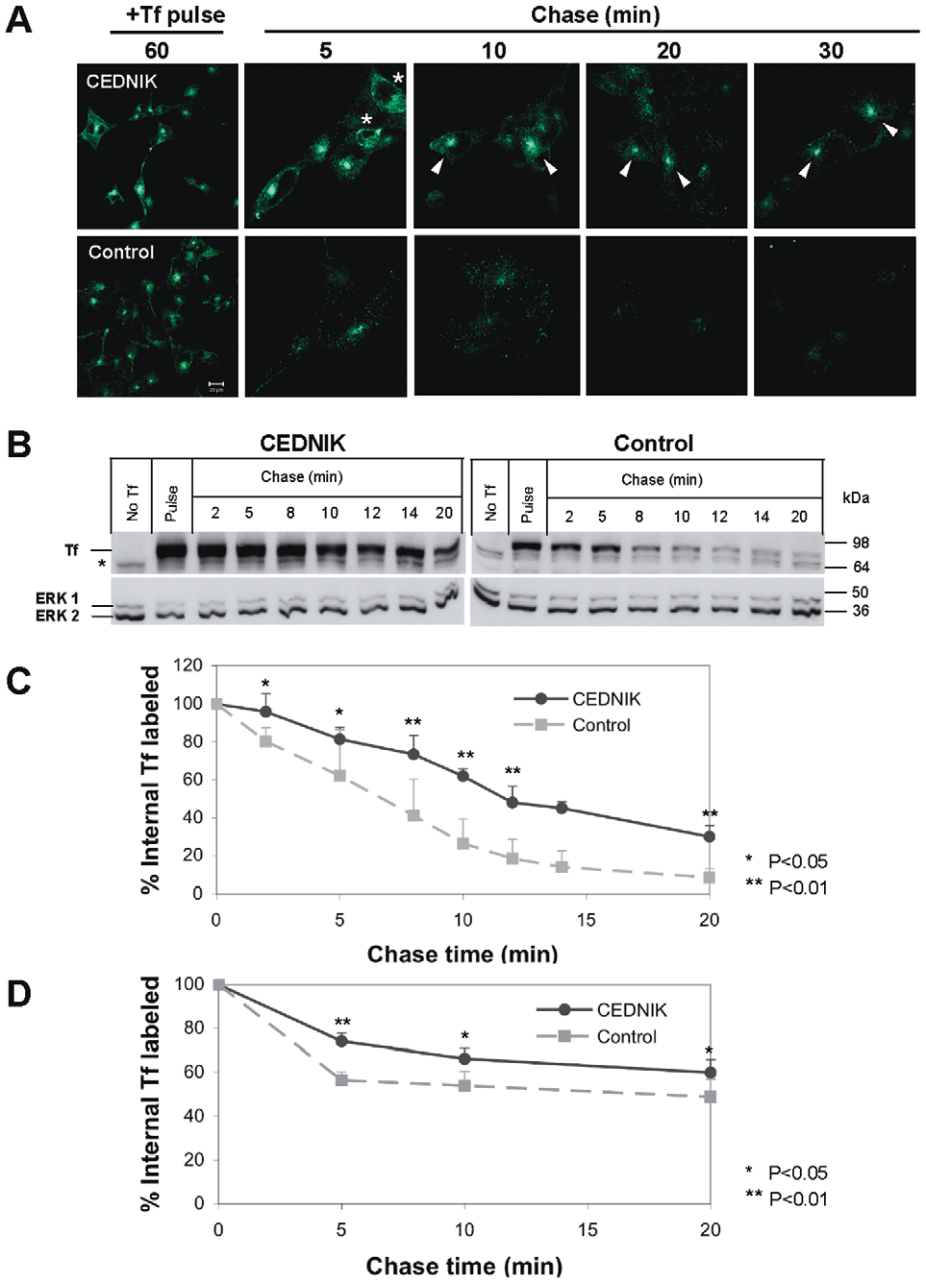
Delayed transferrin recycling in SNAP29 deficient fibroblasts. (A) CEDNIK (F110T) and control (SV80) cells were starved and labeled with AlexaFluor 488-conjugated transferrin. After different times of chase, cells were fixed and visualized by confocal microscopy. Arrows indicate transferrin accumulation in the ERC. Asterisks denote dispersed ERC, as in Figure 4C. (B) CEDNIK and control cells (as in A) were pulsed with biotin-conjugated transferrin and chased for the indicated times. Cell lysates were subjected to Western blot analysis and interaction with HRP-conjugated streptavidin and anti-ERK antibodies, as a loading control. (C) Densitometric analysis of the kinetics of transferrin recycling in CEDNIK and control cells, presented in B. To normalize the results, intensity of the transferrin band at each lane was divided by the intensity of total ERK (ERK1 and ERK2) in the same lane. The value obtained for the pulse time was considered as 100%. (D) Cells were labeled with AlexaFluor 488-conjugated transferrin and then chased for the indicated times after which they were analyzed by flow cytometry. The initial level of internalized transferrin in the pulse was considered as 100%. Tf- transferrin, No Tf- non labeled cells. *- represents non-specific band found in non labeled cells. Bar, 20 µm. Asterisks indicate statistical significance (*p<0.05 and **p<0.01) as analyzed by Student's t-test. Transferrin recycling is attenuated in CEDNIK fibroblasts.

Our results indicated aberrant morphology of several endocytic organelles in CEDNIK cells. We, therefore, tested whether the morphological changes have an impact on endocytosis. For this purpose, transferrin recycling was studied, using pulse-chase assays and flow cytometry (Figure 5). Cells were labeled with AlexaFluor 488-conjugated transferrin for 1 h and monitored during different times of chase. As shown in Figure 5A, transferrin internalization was unchanged in CEDNIK cells compared to control fibroblasts. However, CEDNIK cells displayed an accumulation of transferrin in the perinuclear region (Figure 5A, arrows) and recycling of the ligand was slower than that observed in control cells. Assays with biotinylated transferrin confirmed the impaired endocytic recycling kinetics of this ligand in CEDNIK cells (Figure 5B). During the chase times, disappearance of transferrin from CEDNIK cells was attenuated by 26–30% compared to control cells (Figure 5C). We also quantified the kinetics of transferrin trafficking by flow cytometry-based experiments, which confirmed the attenuation in its endocytic recycling (Figure 5D).

### Recycling of β1-integrin is impaired in SNAP29 deficient cells

**Figure 6:**
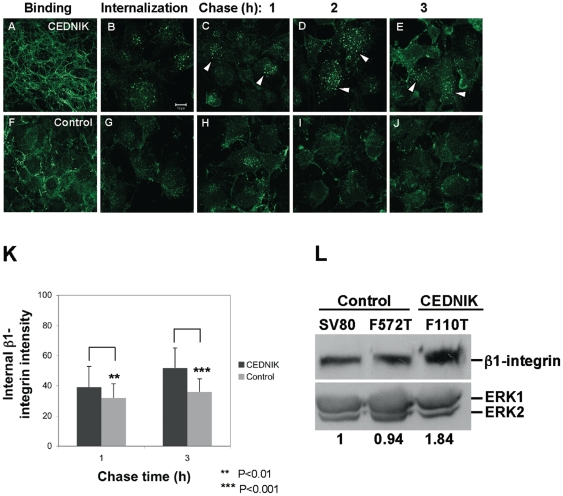
Loss of SNAP29 in fibroblasts causes accumulation of β1-integrin in internal vesicles. (A–J) CEDNIK (F110T) and control (SV80) fibroblasts, grown on fibronectin coated plates, were interacted with anti-β1-integrin antibody (binding: A and F). Following internalization (B, G), cells were acid-washed and chased for the indicated times (C–E, H–J). After all the procedures, cells were fixed and immunostained with AlexaFluor 488-conjugated anti-mouse secondary antibody. Arrows indicate accumulation of β1-integrin in enlarged vesicles in CEDNIK cells. (K) The amount of internalized β1-integrin in CEDNIK and control cells was quantified in 50–70 cells, using the ImageJ program. Asterisks indicate statistical significance (**p<0.01 and **p<0.001) as analyzed by Student's t-test. (L) To test the amount of β1-integrin in the different cells, Western blot analysis was performed and the corresponding blot was interacted with anti-β1-integrin antibody and anti-ERK antibodies, as a loading control. Densitometry was used to quantify the amount of β1-integrin in the different samples (numbers appear under each lane), using the Image Densitometer 1Dprime. To normalize the results, the intensity of the β1-integrin band at each lane was divided by the intensity of ERK in the same lane. The value obtained for SV80 was considered as 1. Bar, 10 µm. Recycling of β1-integrin in CEDNIK cells is attenuated and its amount in these cells is higher compared to control fibroblasts.

Previous studies indicated that inhibition of a SNARE complex component disrupted intracellular trafficking of β1-integrin [28], [29], [30]. Integrins are transmembrane receptors for components of the extracellular matrix (ECM), which provide a link between the ECM and the cytoskeleton. As a consequence, integrins play a role in transmission of signals to the intracellular milieu [31] and undergo endocytic recycling [32]. Integrins play a major role in cell migration, which is a hallmark of epidermal differentiation [33] and brain development [34], [35]. In addition, CEDNIK syndrome is characterized by cerebral dysgenesis, which has been related to defective neuronal migration. We therefore analyzed the trafficking of β1-integrin in CEDNIK fibroblasts (Figure 6). Plasma membrane staining of β1-integrin was more intense in CEDNIK cells compared to control cells exhibiting a fiber network staining (Figure 6A and F). Intracellular endocytic recycling of β1-integrin was assessed using a pulse–chase experiment. CEDNIK cells accumulated β1-integrin containing vesicles (Figure 6C, D and E), which were enlarged and displayed a stronger signal in comparison to control cells (Figure 6H, I and J), indicating a slower trafficking. These findings were confirmed by quantitation of internal β1-integrin. The results showed that in CEDNIK cells, β1-integrin was still retained in vesicles after 3h of chase (Figure 6K). Moreover, results of Western blot analysis on lysates from CEDNIK and control fibroblasts strongly indicated higher β1-integrin levels in CEDNIK cells (Figure 6L).

In CEDNIK as well as in control fibroblasts, plasma membrane β1-integrin rarely colocalized with F-actin, stained with phalloidin (Figure S1A). In addition, we tested a possible colocalization of β1-integrin in SNAP29 containing vesicles. The results indicated occasional localization of β1-integrin in SNAP29 containing vesicles (Figure S1 B, C).

### The distribution of focal adhesion complexes is altered in CEDNIK cells

**Figure 7:**
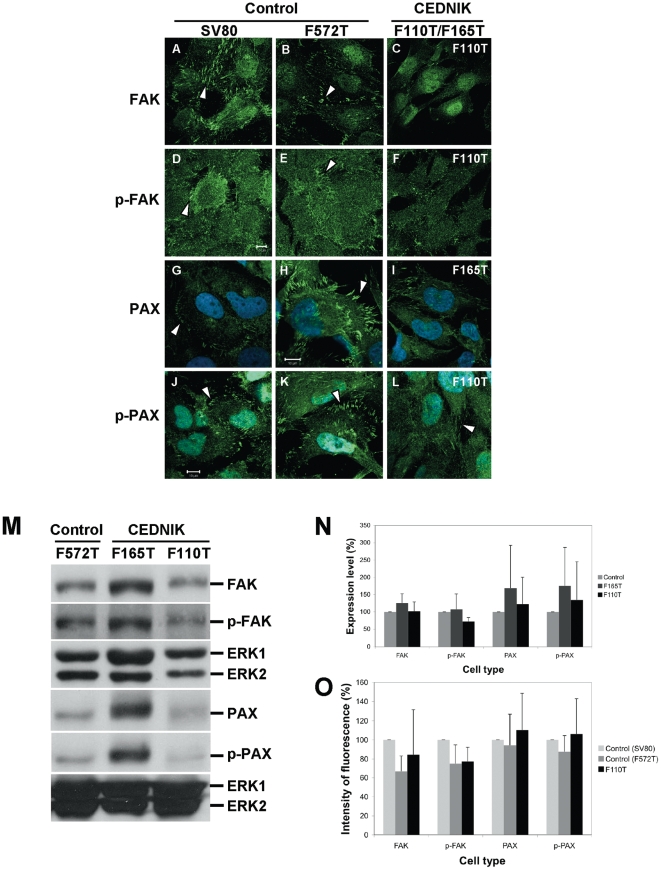
Loss of SNAP29 affects the pattern of focal adhesion complexes. (A–L) Control (SV80 and F572T) and CEDNIK (F110T or F165T) fibroblasts were fixed and stained with anti-FAK (A–C), anti-p-FAK (D-F), anti-PAX (G–I) and anti-p-PAX antibodies (J–L). Nuclei were stained with Hoechst. Bar, 10 µm. (M) Western blot analysis showing the expression of FAK, p-FAK, PAX and p-PAX in control (F572T) and CEDNIK (F165T, F110T) cells. Anti-ERK antibodies were used as a loading control. The blot is a representative of 5 independent experiments. (N) Bar graph illustrating the expression levels of the focal adhesion components: FAK, p-FAK, PAX and p-PAX. Densitometry was used to quantify the amount of each focal adhesion component as seen in Western blot, using the Image Densitometer 1Dprime. To normalize the results, the intensity of each band was divided by the intensity of ERK in the same lane. The value obtained for control cells (F572T) was considered as 100%. (O) Bar graph showing quantitation of the intensity of FAK, p-FAK, PAX and p-PAX staining, measured in 14-168 cells, using the ImageJ software. The value obtained for control cells (SV80) was considered as 100%. Expression pattern of FAK, phospho-FAK, paxillin and phospho-paxillin is different in CEDNIK cells in comparison to control cells. However, their total level is similar.

Focal adhesion kinase (FAK), paxillin (PAX) and their phosphorylated forms (p-FAK and p-PAX, respectively) are integral components of the focal adhesion complexes [36]. Since impaired recycling of β1-integrin affects cell motility [31], we tested whether there are structural abnormalities of the focal adhesion proteins in CEDNIK cells. Our results indicated decreased FAK and phosphorylated-FAK expression in peripheral adhesions in CEDNIK cells, in comparison to control fibroblasts (Figure 7A–F). Staining for PAX and phoshorylated-PAX disclosed thinner focal adhesion structures and diffuse cytoplasmic distribution in CEDNIK fibroblasts in comparison to control fibroblasts (Figure 7G–L). Albeit the structural abnoramalities no significant changes were observed at the total protein level as measured in Western blots (Figure 7M, N) or in immunofluorescence images (Figure 7O).

Taken together, these results provide evidence for structural abnormalities in focal adhesion complexes in CEDNIK fibroblasts.

### Loss of SNAP29 affects cell migration and cell spreading

**Figure 8:**
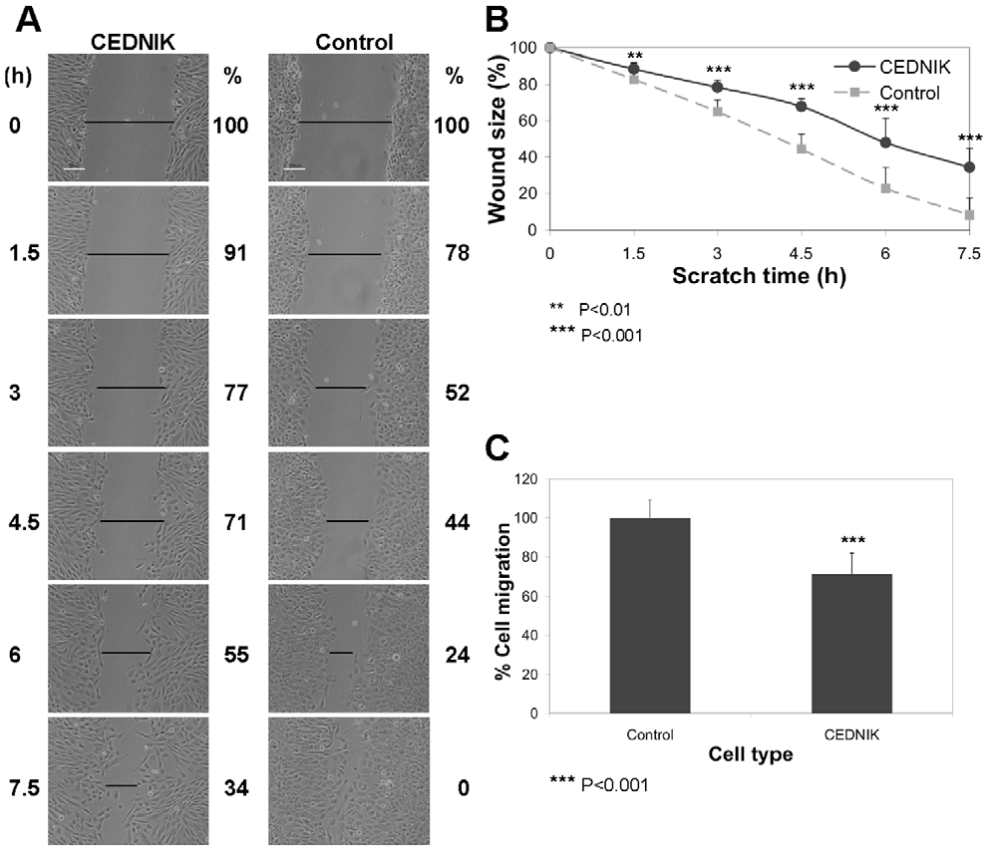
Loss of SNAP29 impairs cell migration. (A) Confluent CEDNIK (F110T) and control (SV80) fibroblasts were plated on fibronectin coated plates. Scratches, represented by the horizontal black bars, were introduced and measured. Cells were allowed to migrate and were visualized under a microscope every 1.5 h. (B) A graph, showing the kinetics of cell migration. The results represent 8 independent experiments. The initial size of the wound at time 0 was considered as 100%. (C) Bar graph presenting the colonization capacity of the cells (% cell migration) after 7.5 h, measured in eight independent experiments. Control cells were considered as 100%. White bar, 60 µm. Asterisks indicate statistical significance (**p<0.01 and ***p<0.001) as analyzed by Student's t-test. The results indicate retarded migration of CEDNIK cells.

Recycling of β1-integrin is essential for cell motility [37]. Given the observed impaired recycling of β1-integrin, its accumulation in enlarged endosomes in CEDNIK cells (Figure 6) and the distinguishable expression pattern of FAK, PAX and their phosphorylated forms in peripheral adhesions (Figure 7), we examined the ability of CEDNIK cells to migrate and spread using wound healing and cell spreading assays. Following wound, control fibroblasts, grown on fibronectin, which engages β1-integrin, migrated into the scratch area and closed the wound after 7.5 h (Figure 8A and B). CEDNIK fibroblasts showed a retarded cell migration (Figure 8A and B), exhibiting 29% reduction in the overall migration rate after 7.5 h (Figure 8C).

**Figure 9:**
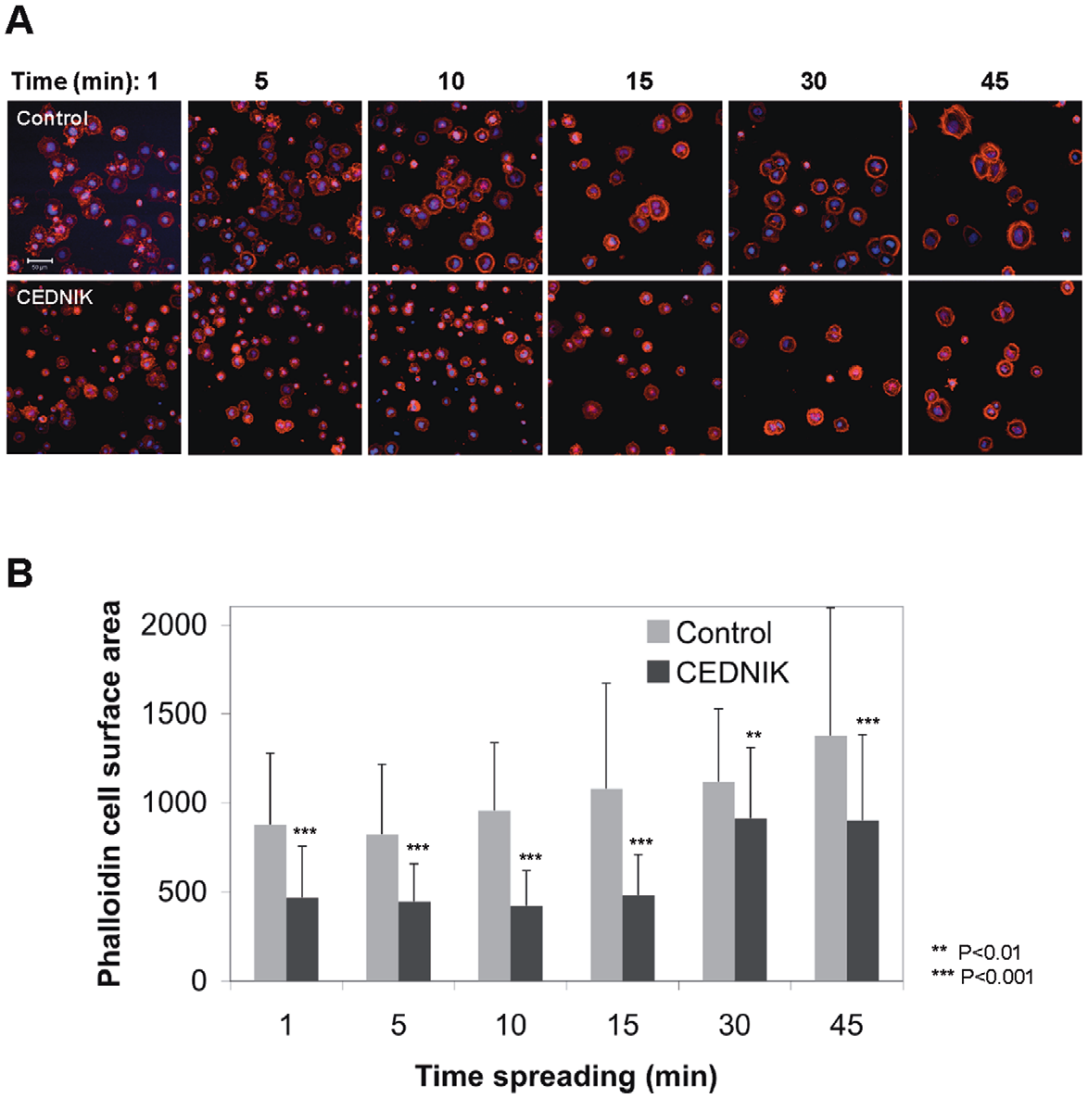
Cell spreading is affected in SNAP29 deficient cells. (A) Confluent CEDNIK (F110T) and control (SV80) fibroblasts were grown in fibronectin coated plates. Following trypsizination, cells were allowed to spread for the indicated times, fixed, permeabilized and stained with AlexaFluor 568-conjugated phalloidin. Nuclei were stained with Hoechst. (B) Quantitative analysis of cell surface area of individual cells was performed using the ImageJ program. Bar, 50 µm. Asterisks indicate statistical significance (**p<0.01 and ***p<0.001) as analyzed by the Student's t-test. Spreading of CEDNIK cells is retarded compared to control cells.

We also examined whether lack of SNAP29 interferes with cell spreading. Cells were allowed to spread on fibronectin, following trypzinization. Cell surface staining was quantified in phalloidin-labeled cells. The results indicated that spreading of CEDNIK cells was significantly slower than that of control cells (Figure 9A and B). While control cells started to spread 10 min after seeding, CEDNIK cells had a lag of 15 min and their spreading rate was lower than that of control cells (Figure 9B).

To summarize, our results showed that CEDNIK cells display decreased motility, exemplified by attenuated rate of wound healing and cell spreading, highlighting the importance of SNAP29 mediated membrane fusion in these processes.

## Discussion

SNAP proteins are t-SNAREs that participate in fusion processes during endocytosis and exocytosis [3], [38]. Ample work has been published on the importance of SNAP25, a neuronal SNAP, in regulating neurotransmitter release and modulating calcium dynamics in response to depolarization [39], [40]. SNAP23 is required for exocytosis from platelet granules [6]. The importance of SNAP29 in fusion processes has not been extensively studied. The discovery that CEDNIK syndrome results from absence of SNAP29 provided us with the means to study the biological role of this protein.

In this work, we present results strongly indicating that ablation of SNAP29 leads to impaired recycling of clathrin-dependent (transferrin and β1-integrin) and clathrin independent (β1-integrin) ligands. It also leads to changes in the architecture of EEA1 containing early endosomes, Rab11-expressing domains of the endocytic recycling compartment and the Golgi complex. We further show that the inability of SNAP29 deficient cells to recycle β1-integrin affects the structure of focal adhesions as exemplified by changes in the membranal expression pattern of FAK, p-FAK, PAX and p-PAX, which are integral components of the focal adhesion complexes. Such changes in the structure of focal adhesion complexes could explain the defects in cell migration observed in CEDNIK fibroblasts. Higher β1-integrin levels were observed at the plasma membrane in CEDNIK cells compared to control cells. Moreover, quantitation analyses revealed that the level of steady state β1-integrin is also increased in CEDNIK cells. We assume that this elevation reflects impaired trafficking of β1-integrin containing vesicles from the plasma membrane to the lysosomes, where β1-integrin undergoes degradation.

β1-integrin, as a member of the integrin family, is a transmembrane receptor for components of the extracellular matrix. It functions in a wide variety of processes including: migration, proliferation, differentiation, apoptosis and the regulation of focal adhesion complexes. Regarding motility, β1-integrin recycles to the leading edge of the cell during cell migration to facilitate contact with the ECM, thereby stabilizing lamellipodia and supporting the generation of motile forces [31], [41]. Its clustering promotes FAK phosphorylation, which creates a binding site for the Src-homology SH2 domain. This FAK-Src complex facilitates SH3-mediated binding of the adaptor protein p130Cas (breast cancer anti-estrogen resistance protein 1) to FAK and its subsequent phosphorylation. Crk (sarcoma virus c-10 oncogene homology) binding to phosphorylated p130Cas facilitates Rac (related to A and C protein kinases) activation, lamellipodia formation and cell migration [42]. Absence of several components in the β1-integrin signaling pathway leads to cell spreading defects and refractory cell motility responses. Thus, fibroblasts that derived from either FAK^−/−^, p130Cas^−/−^ or paxillin^−/−^ mice exhibited retarded integrin-stimulated migration [42]. Paxillin, an integrin-binding protein recruits FAK and vinculin to focal contacts [42]. Disruption of integrin endocytosis and recycling impaired cell spreading and migration [43]. It is worth noting that involvement of SNARE-mediated trafficking in cell migration and β1-integrin traffic has been reported in cells expressing a dominant negative form of NSF (E329Q-NSF) or through inhibition of the v-SNARE cellubrevin/VAMP3 (vesicle-associated membrane protein 3) by the catalytic light chain of tetanus toxin and by its silencing [28], [29], [30].

Migration is a crucial step in epidermal stratification and, therefore, in epidermis differentiation and maintenance during adult life [44], [45]. Our results showing defects in cell migration in CEDNIK fibroblasts highlight the importance of membrane fusion mediated by SNAP29 for normal terminal differentiation of the skin. Cell migration is also essential in brain development, and the migration defects in CEDNIK derived fibroblasts could explain the cerebral dysgenesis in the patients, manifested by polymicrogyria (excessive number of small convolutions (gyri) on the surface of the brain) and pachygyria (thick convolutions of the cerebral cortex) which are both disorders of neuronal migration [46].

SNAP29 may not be the only SNAP protein associated with neurological abnormalities. Recently, an association between the abnormal expression of SNAP25 and bipolar disease has been reported [47]. Furthermore, a loss of SNAP25 was noted in brains of patients suffering from Huntington's disease [48].

We did not observe a trafficking defect to and from the Golgi in CEDNIK fibroblasts, though Golgi architecture was abnormal. It is still plausible that secretion of proteins and lipids is impaired in CEDNIK keratinocytes, as previously shown [14]. Interestingly, there are cutaneous diseases, in which defective exocytosis from the Golgi apparatus has been demonstrated. Thus, ARC (arthrogryposis-renal dysfunction-cholestasis) syndrome is caused by mutation in the SNARE protein VPS33B, whose yeast homolog was shown to have a key role in late stages of protein trafficking from the Golgi to the vacuole [49]. In Cutis Laxa Type 2, caused by a loss of the a2 subunit of the vesicular ATPase H^+^-pump, fibroblasts showed distended Golgi cisternae, impaired secretion and increased intracellular retention of tropoelastin [50]. In Harlequin syndrome, characterized by disturbance in lamellar granule, ichthyosis and severe genodermatosis, ABCA12, an ATP-binding cassette transporter, is mutated. This leads to impaired lipid transport from the Golgi to lamelar granules in keratinocytes [51].

In conclusion, our results strongly indicate that in CEDNIK fibroblasts there is compromised recycling and, therefore, intracellular accumulation of transferrin and β1-integrin. Impaired recycling of β1-integrin leads to retarded cell motility and spreading.

## Materials and Methods

### Cells and transfection

SNAP29 deficient primary fibroblasts, derived from two CEDNIK patients (F110 and F165), were transformed with Simian Virus 40 (SV40) large T-antigen (F110T and F165T) as previously described [14]. SV80 (SV40 transformed normal human fibroblasts) [52] and normal fibroblasts transformed with SV40 large T-antigen (F572T) were used as controls. Cells were cultured in Dulbecco's Modified Eagle medium (DMEM) supplemented with 10% Fetal calf serum (FCS; Beit Haemek, Israel). Cells were transfected with Fugene 6 reagent (Roche, USA) according to the manufactorer's recommendations.

### Plasmids

GFP-EHD1 [20], GFP-EHD3 [24], YFP-EHD2 and YFP-EHD4 were previously constructed in the lab. To construct the GFP-SNAP29 plasmid, a 1.6 kb fragment of human SNAP29 cDNA was cloned into the *BglII* and *SalI* sites of pEGFP vector (Clontech Laboratories, CA, USA). VSVG-YFP [53] and GalT-YFP were kindly provided by Dr. K. Hirschberg (Tel Aviv University, Israel). Rab11-YFP was kindly provided by Dr. A. Sorkin (University of Colorado Denver, USA).

### Antibodies and ligands

Anti-EHD1 [20] and anti-SNAP29 [12] antibodies were described elsewhere. Anti-ERK (sc-93) and anti-FAK antibodies (sc-558) were from Santa Cruz Biotechnology (Santa Cruz, CA, USA). Horseradish peroxidase (HRP) or Cy2 or Cy3-conjugated goat anti-rabbit or goat anti-mouse IgG were from Jackson ImmunoResearch (West Grove, PA, USA). AlexaFluor 488 (T13342) or biotin conjugated-transferrin (T23363), AlexaFluor 568-conjugated phalloidin (A12380), AlexaFluor 555-conjugated CTxB (B subunit of Cholera Toxin, C-34776), AlexaFluor 488-conjugated goat anti mouse, anti-phospho FAK (44-624G) and anti-phospho-PAX (44-722G) antibodies were from Invitrogen/Molecular Probes (Eugene, OR, USA). HRP-conjugated streptavidin (S5512) and anti-GM130 antibodies (G7295) were from Sigma-Aldrich (Saint Louis, MO, USA). Anti-integrin β1 antibody (anti-human CD29, MCA2028) was from AbD-Serotec (Oxford, England). Anti-EEA1 (610456) and anti-PAX (610052) antibodies were from BD Transduction Laboratories (San Jose, CA, USA). Mouse monoclonal anti-α-chain of AP2 antibody was a gift from Dr. M.S. Robinson (Cambridge Institute for Medical Research, University of Cambridge, UK).

### Immunoblotting

Cells were harvested and lysed in lysis buffer (10 mM Hepes, 100 mM NaCl, 1 mM MgCl_2_, 0.5% NP-40, nonylphenoxylpolyethoxylethanol) containing protease inhibitors (1 mM PMSF- phenylmethanesulphonylfluoride, 1 mg/ml aprotinin and leupeptin; Sigma, St Louis, MO, USA). Following centrifugation at 10,000 *g* for 15 min at 4*°*C, the supernatant was electrophoresed through a 10% SDS-PAGE and transferred to a nitrocellulose membrane (Schleicher Schuell, Dassel, Germany). Membranes were blocked with 5% skim milk in TBS-Tween (20 mM Tris HCl, 4 mM Tris base, 140 mM NaCl, 1 mM EDTA, 0.1% Tween-20) and interacted with the appropriate antibodies after which they were incubated with the secondary antibody and washed with TBS-Tween. Bound antibodies were detected by ECL-Luminol Reagent (Santa Cruz Biotechnology, Santa Cruz, CA, USA). The blots were reprobed with anti-ERK antibodies as a loading control.

### Reverse transcription-polymerase chain reaction (RT-PCR) and quantitative real-time PCR (qRT-PCR)

Total RNA was extracted from fibroblasts using the EZ-RNA isolation kit (Biological Industries, Beit Haemek, Israel). Two to five μg RNA were reverse transcribed in 25 µl, using 100 units of M-MLV reverse transcriptase (Promega, Madison, WI, USA). The resulting cDNA was used to amplify different human Ehds with specific primers. For Ehd2: sense primer 5′-GACGCCACAAGGGCTCCG-3′; antisense primer 5′-CTATGGTAATGCCCAGTGCC-3′; for Ehd3: sense primer 5′-ATGTTCAGCTGGCTGGGTAC-3′; antisense primer 5′-CTCGTGGTTCTTGAGGGC-3′; for Ehd4: sense primer 5′-GGAAGTCCCTGCCCAAGG-3′; antisense primer 5′-CTGCCTCCAGGTGCCCTC-3′. Thermal cycling consisted of 94°C for 10 min, followed by 30 cycles of denaturation (94°C, 1 min), annealing (60°C, 1 min) and extension (72°C, 1 min), and a final extension at 72°C for 10 min.

For qRT-PCR, PCR amplification of cDNA was performed using the Absolute QPCR SYBR Green Mix (ABgene, Epsom, UK) in a Rotor-gene 3000 multifilter system (Corbett Research, UK) with primer pairs specific for Ehd1 (sense primer 5′-GTACCACACAGCTGGGCTTCCC-3′; antisense primer 5′-CGCATCCATCCTCACCTAATAC-3′) and glyceraldehyde 3-phosphate dehydrogenase, (GAPDH; sense primer 5′-GAGTCAACGGATTTGGTCGT-3′; antisense primer 5′-GACAAGCTTCCCGTTCTCAGCC-3′). Cycling conditions were as follows: 95***°***C for 10 min: 95***°***C for 10 s, 60***°***C for 25 s and 72***°***C for 15 s for a total of 40 cycles and one cycle of 95***°***C for 30 s, 60***°***C for 30 s and 95***°***C for 30 s. Each sample was analyzed in triplicate. For quantitation, standard curves were obtained using serially diluted cDNA, amplified in the same real-time PCR run. Results were normalized to GAPDH mRNA levels.

### Immunocytochemistry

Cells were plated on coverslips in 24-well plates. Cultures were washed with PBS and fixed with 4% paraformaldehyde in PBS at 4°C for 15 min. Following washes, the cells were incubated in 3% BSA and 0.1% triton X-100 (Sigma-Aldrich, Saint Louis, MO, USA) in PBS for 10 min, after which they were incubated with the respective primary antibodies for 1 h. Following washes, cell cultures were incubated with the respective secondary antibodies. Cells were washed, mounted with galvanol and imaged in Confocal Zeiss LSM 510 or LSM 510 Meta microscopes (Carl Zeiss, NY, equipped with an argon laser).

### VSVG trafficking

Subconfluent cultures grown in 24 well plates were transfected with 1 µg of VSVG-YFP ts045 (a temperature sensitive mutant of the VSVG protein) and incubated at 40°C for 18 h. To follow VSVG transport through the secretory pathway, cells were transferred to 37°C and at different times of chase they were fixed with 4% paraformaldehyde and imaged using confocal microscopy.

### Cholera toxin trafficking

Cells, cultured on coverslips coated with 20 µg/ml fibronectin in DMEM without phenol red, supplemented with 10% FCS, were labeled with 0.5 µg/ml AlexaFluor 555-conjugated CTxB at 37°C in DMEM lacking serum but containing 0.1% BSA, 20 mM Hepes, pH 7.2. Cells were mounted with galvanol for confocal microscopy.

### Uptake and recycling of transferrin

For confocal analysis, fibroblasts were grown on coverslips and were serum-starved for 30 min in binding medium (DMEM, 0.1% BSA, 20 mM Hepes, pH 7.2, no phenol red). Following incubation with 25 µg/ml Alexa 488-conjugated transferrin at 37***°***C for 1 h, cells were rapidly chilled on ice and washed three times with cold PBS. Chase was performed for different times with media containing serum (20% dialyzed FCS, 20 mM Hepes pH 7.2, 50 mM deferoxamine, 2.5 mg/ml holo-transferrin) at 37***°***C. Cells were washed with cold PBS, fixed with 4% paraformaldehyde and mounted with galvanol mounting solution for confocal microscopy.

Kinetics of transferrin recycling was performed in cells grown on 3 cm plates and serum-starved for 30 min in binding medium as described above. Following 15 min incubation with 20 µg/ml of biotinylated transferrin at 37***°***C, cells were chilled on ice and washed once with cold citric buffer medium (25.5 mM citric acid pH 3, 24.5 mM sodium citrate, 280 mM sucrose, 0.01 mM deferoxamine) and twice with cold PBS. Chase was performed with media containing serum (20% dialyzed FCS, 20 mM Hepes pH 7.2, 50 mM deferoxamine, 2 mg/ml holo-transferrin) at 37***°***C. Following several washes with cold PBS, cells were processed as described under immunoblotting. Samples, containing 20 µg of protein, were electrophoresed through a 10% SDS-PAGE and transferred onto a nitrocellulose membrane (Schleicher and Schuell BioScience, Keene, NH, USA). The corresponding blots were blocked in a TGG solution (50 mM Tris pH 7.4, 100 mM NaCl, 1 M glucose, 10% glycerol, 0.5% Tween-20) with 1% skim milk and 3% BSA, for 1 h at 4***°***C. This was followed by one wash with TGG and two additional washes with TBS-Tween-20. Blots were probed with streptavidin/peroxidase for 1 h and proteins were detected using the ECL-Luminol Reagent. Membranes were rebloted with anti-ERK antibodies as a loading control. Blots were scanned and the intensity of the proteins was measured by the image densitometer 1Dprime (Amersham Pharmacia Biotech, USA).

### Flow cytometry analysis

Cells were serum starved in DMEM containing 0.1% BSA, 20 mM Hepes, pH 7.2 for 30 min. Twenty five μg/ml of human Alexa 488-conjugated transferrin was added for 15 min at 37***°***C. After three washes with PBS, cells were replenished in media containing 20% dialyzed FCS, 20 mM Hepes pH 7.2, 50 mM deferoxamine and 2.5 mg/ml holo-transferrin. Following different incubation times at 37***°***C, they were washed with PBS, removed from the dish with warm trypsin (15 s treatment), transferred to 4 ml cold DMEM and pelleted by centrifugation. Cell pellets were resuspended in 500 µl of 4% paraformaldehyde. Alexa-488 conjugated transferrin was followed by flow cytometry in 5,000 cells using a Becton Dickinson FACSort (Mountain View, CA) and CellQuest software. Baseline staining was obtained using non-labeled cells.

### β1-integrin recycling

To follow surface β1-integrin, cells were cultured on coverslips, coated with 20 µg/ml fibronectin, The cells were incubated in DMEM without phenol red, supplemented with 10% FCS and starved for 1 h at 37°C in DMEM lacking serum but containing 0.5% BSA before addition of 5 µg/ml of anti-β1-integrin antibody for 1 h on ice. For internalization, after the above treatment, cells were transferred to 37°C for an additional 1 h to allow receptor-antibody internalization. Surface antibodies were removed by acid washes (0.5% acetic acid, 0.5 M NaCl, pH 3.0). Chase was performed in complete media containing 20% FCS for different times after which cells were fixed with 4% paraformaldehyde. Cells were stained with AlexaFluor 488-conjugated goat anti-mouse secondary antibody for 1 h and mounted with galvanol for confocal microscopy. Internal β1-integrin was measured in 50–70 cells, chosen randomly for each time point, using ImageJ software.

### Cell migration assay

Cells were grown at equivalent confluence for 18 h on 6 well plates, precoated with 20 µg/ml fibronectin. Scratches were introduced with a thin pipette tip, and cells were allowed to migrate into the wound at 37°C. Pictures were taken every 90 min with a Nikon microscope (Eclipse TE 2000-S, Japan). The size of the scratch at time zero was considered 100%.

### Cell spreading assay

Cells were trypsinized and replated on coverslips precoated with 20 µg/ml fibronectin at a concentration of 20,000 cells/well. The cells were allowed to spread for different times after which they were fixed and labeled with 0.2 units/ml of AlexaFluor 568-conjugated phalloidin and 1 µg/ml of Hoechst (Sigma-Aldrich, Saint Louis, MO, USA) for 1 h. Cell surface boundaries were outlined for 60–280 cells, chosen randomly for each time point, and ImageJ software was used to calculate the surface area of each cell.

## Supporting Information

Figure S1 Colocalization of β1-integrin with F-actin and SNAP29. (A) CEDNIK (F110T) and control (SV80) fibroblasts were grown on fibronectin coated plates and interacted with anti-β1-integrin antibody at 4°C for 1 h on ice. Cells were washed, fixed, permeabilized and immunostained with AlexaFluor 488-conjugated anti-mouse secondary antibody and AlexaFluor 568-conjugated phalloidin. Nuclei were stained with Hoechst. Cells were examined under a confocal microscope. Arrows indicate colocalization of β1-integrin and phalloidin stained F-actin. (B) Control and CEDNIK fibroblasts (as above) were transfected with GFP-SNAP29 expressing plasmid. Eighteen h later β1-integrin binding assay was performed and was followed by internalization for 1 h and a 2 h chase, as detailed in Materials and Methods. The cells were fixed, and immunostained with secondary Cy3-conjugated anti-mouse antibody. (C) Binding, internalization and chase of β1-integrin were performed in control (SV80) fibroblasts as above, after which the cells were fixed and immunostained with secondary AlexaFluor 488-conjugated anti-mouse antibody. Endogenous SNAP29 was interacted with anti-SNAP29 antibodies and stained with secondary Cy3-conjugated goat anti rabbit antibodies. Nuclei were counterstained with Hoechst. Bar, 50 µm. Arrows in (B, C) indicate colocalization between β1-integrin and SNAP29. β1-integrin shows a plasma membrane staining and is rarely colocalized with F-actin. There is occasional β1-integrin staining in SNAP29 containing vesicles.
(4.88 MB TIF)
