# Supplementary figures and images for: Loss of SNAP29 Impairs Endocytic Recycling and Cell Motility
Source: PLoS One. 2010 Mar 18;5(3):e9759. doi: 10.1371/journal.pone.0009759 (PMC2841205; doi:10.1371/journal.pone.0009759)

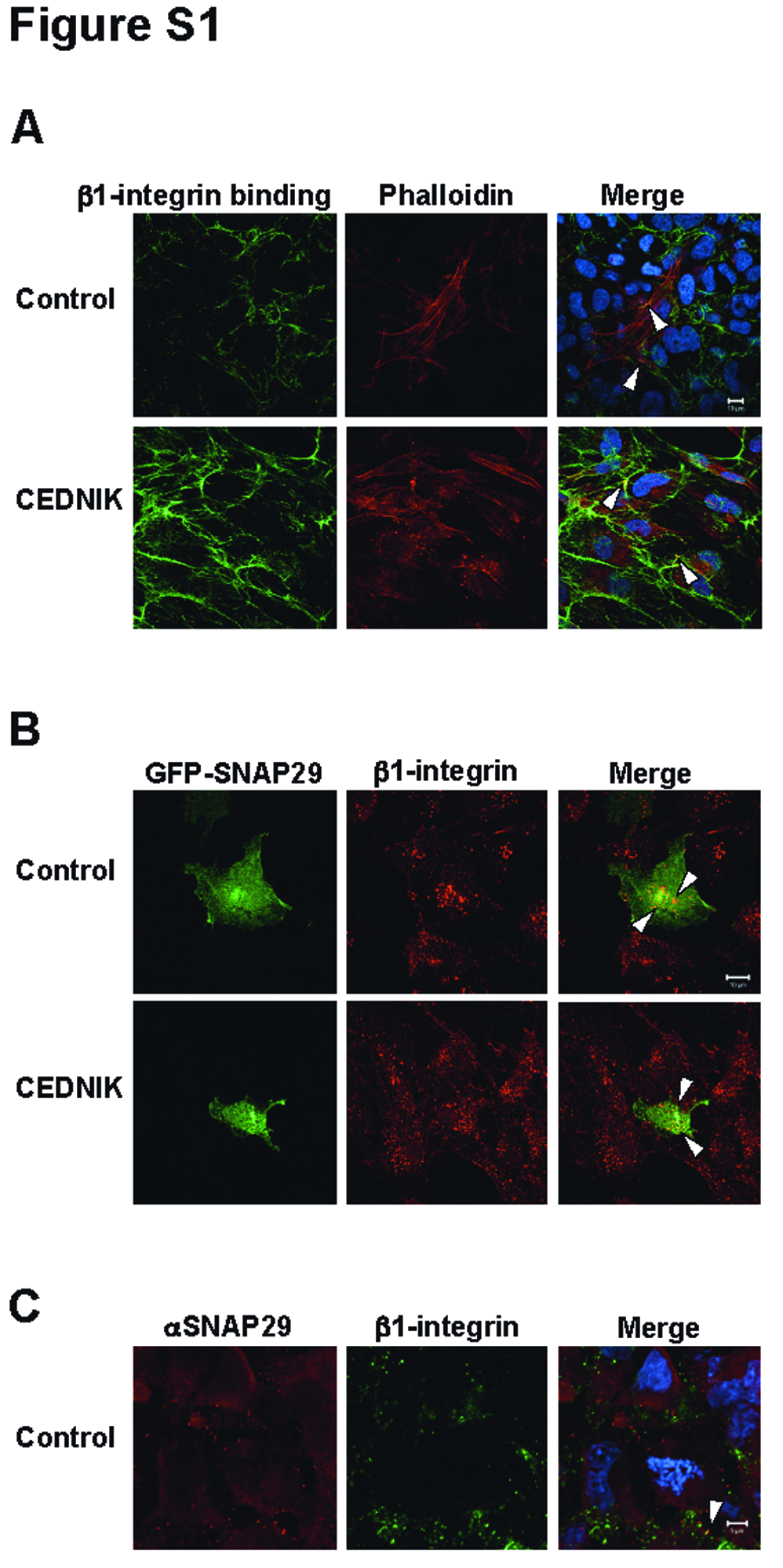

Supplement: Figure S1 — Colocalization of β1-integrin with F-actin and SNAP29. (A) CEDNIK (F110T) and control (SV80) fibroblasts were grown on fibronectin coated plates and interacted with anti-β1-integrin antibody at 4°C for 1 h on ice. Cells were washed, fixed, permeabilized and immunostained with AlexaFluor 488-conjugated anti-mouse secondary antibody and AlexaFluor 568-conjugated phalloidin. Nuclei were stained with Hoechst. Cells were examined under a confocal microscope. Arrows indicate colocalization of β1-integrin and phalloidin stained F-actin. (B) Control and CEDNIK fibroblasts (as above) were transfected with GFP-SNAP29 expressing plasmid. Eighteen h later β1-integrin binding assay was performed and was followed by internalization for 1 h and a 2 h chase, as detailed in Materials and Methods. The cells were fixed, and immunostained with secondary Cy3-conjugated anti-mouse antibody. (C) Binding, internalization and chase of β1-integrin were performed in control (SV80) fibroblasts as above, after which the cells were fixed and immunostained with secondary AlexaFluor 488-conjugated anti-mouse antibody. Endogenous SNAP29 was interacted with anti-SNAP29 antibodies and stained with secondary Cy3-conjugated goat anti rabbit antibodies. Nuclei were counterstained with Hoechst. Bar, 50 µm. Arrows in (B, C) indicate colocalization between β1-integrin and SNAP29. β1-integrin shows a plasma membrane staining and is rarely colocalized with F-actin. There is occasional β1-integrin staining in SNAP29 containing vesicles. (4.88 MB TIF) [file pone.0009759.s001.tif]
